# A Retrospective Multisite Case-Control Series of Concomitant Use of Daptomycin and Statins and the Effect on Creatine Phosphokinase

**DOI:** 10.1093/ofid/ofz444

**Published:** 2019-11-07

**Authors:** Bethany Lehman, Elizabeth A Neuner, Victor Heh, Carlos Isada

**Affiliations:** 1 Department of Infectious Diseases, Cleveland Clinic, Cleveland, Ohio, USA; 2 Department of Pharmacy, Barnes-Jewish Hospital, St. Louis, Missouri, USA; 3 CORE Research Office, Ohio University Heritage College of Osteopathic Medicine, Dublin, Ohio, USA

**Keywords:** CPK elevation, daptomycin, myalgia, statin

## Abstract

**Objective:**

Daptomycin has been associated with increased creatine phosphokinase (CPK) due to muscle injury leading to myalgias and muscle weakness. Statins have been proven to cause the same effects and it is recommended to discontinue the use of statins while on daptomycin. Evidence regarding this drug interaction is mixed. This study evaluated the risk of CPK elevation in concomitant use of daptomycin and statins compared to daptomycin alone.

**Method:**

This is a multisite retrospective case-control study of patients who received daptomycin therapy with monitoring of CPK. Rates of CPK elevations were compared in patients receiving daptomycin with a statin versus daptomycin alone. To estimate the association between CPK elevation and daptomycin therapy controlling for other risk factors, logistic regression was used to analyze data. Statistical significance was determined at ɑ of 0.05.

**Results:**

A total of 3658 patients were included in the study, with 2787 on daptomycin therapy alone and 871 with concurrent statin use. The incidence of CPK elevation was 90 events (3.2%) in the daptomycin group and 26 events (3.0%) in the concurrent statin group. Patients who received daptomycin therapy in addition to statins had no statistically significant difference from patients on daptomycin alone (hazard ratio, 1.05; *P* = .85; 95% confidence interval, 0.61–1.84). After adjusting for potential risk factors, the hazards ratio remained almost the same.

**Conclusions:**

Concomitant use of daptomycin and statin did not show an increase risk of CPK elevation. Clinicians may consider concomitant use of daptomycin and statin therapy with weekly CPK monitoring.

## INTRODUCTION

Daptomycin is a cyclic lipopeptide bactericidal antibiotic that is used in Gram-positive infections, such as those caused by *Staphylococcus aureus*, including methicillin resistant *Staphylococcus aureus*, *Streptococcus pyogenes*, and vancomycin resistant *Enterococcus*. Daptomycin has been associated with an increase in creatine phosphokinase (CPK) due to muscle injury leading to myalgias and muscles weakness [1, 2]. The mechanism of muscle injury is incompletely understood. Manufacturers recommend that CPK is monitored at the beginning of therapy and at least weekly in all patients taking daptomycin [3]. Statins, or 3-Hydroxy-3-methyl-glutaryl coenzyme A (CoA) reductase inhibitors, are used for cholesterol and lipid lowering by blocking the formation of cholesterol by the liver. Statins have been associated with an increase in CPK, myalgias, and myositis [4]. According to the drug manufacturing information for Cubicin (daptomycin), one should consider suspending statins while using daptomycin [3]. However, withholding statin therapy may have negative neurologic or cardiac effects on patients, including mortality, especially for those suffering acute stroke or recent myocardial infarction [5, 6]. The literature associated with the concern of elevated CPK in patients on both daptomycin and statin therapy is small and has mixed results as these studies have limitations.

The primary objective of this study is to determine if there is an increased incidence of elevated CPK in patients taking daptomycin plus a statin over daptomycin alone.

## METHODS

An institutional review board approved this multisite retrospective case-control study that was performed by chart review of patients at the Cleveland Clinic Main Campus and 8 smaller Cleveland Clinic community hospitals from April 2004 to April 2016. Cases were identified by electronic health records of daptomycin pharmacy administration codes. Manual individual chart review was not performed on all patients. Outpatient data were able to be captured as daptomycin is administered by an outpatient parenteral antibiotic therapy (OPAT) team ordered with home health care. Outpatient labs from the OPAT team are recorded in the electronic medical record. Male and female patients 18 years and older who had been on daptomycin for over 72 hours, as either inpatient or outpatient, were included. Patients who received multiple courses of daptomycin were included as well. Patients were excluded if they had an elevated CPK at baseline, were missing follow up CPKs, or did not have at least 2 CPKs completed during therapy. The case group was on daptomycin and statin therapy, which was compared with a control group of patients on daptomycin alone. Statin use was identified by pharmacy administration codes. Patients were considered in the concurrent statin group if they were administered a statin during the period that daptomycin was administered. This included patients whose statins were discontinued while on daptomycin in accordance with the time-varying covariate analysis. If a patient was on a statin prior, but the statin was held for a time of daptomycin administration, then the patient was considered to be in the daptomycin-alone group. Elevation of CPK was defined as elevation at 5 times the upper normal limit, which was >1000 U/L for this study. In the adjusted analysis, sex, race, and body mass index (BMI) were controlled. Other potential causes of CPK elevation, including recent surgery, cancer, myocardial infarction, and chronic kidney disease, were collected and controlled for as well. Recent surgery was defined as a surgery performed within 90 days of daptomycin administration. Diagnosis codes for rhabdomyolysis and myalgias were collected during the course of daptomycin to monitor for complications. Comorbidities were identified by International Classification of Diseases (ICD) codes except obesity that was defined by BMI ≥ 30. The transplantation group included both solid organ transplant and bone marrow transplant patients. Skin cancer was excluded from the cancer patients list.

### Statistical Analysis

Summary statistics such as mean, median, proportion or percentages, standard deviation, and interquartile range were used to summarize data for inclusion. Kaplan-Meier techniques were used to determine incidence of CPK elevation since first daptomycin order. Cox regression models were used to determine the hazards of CPK elevation between those on daptomycin alone and those on both daptomycin and statin and to control for other risk factors. The medication group (daptomycin alone or both daptomycin and statin) was treated as a time-varying covariate in order to determine medication status within time intervals they actually occurred. Time-varying covariates are those that may change in value over time. The daptomycin and statin group was treated as a time-varying covariate in order to determine medication status within time intervals they actually occurred. Groups of patients who started as concurrent only remained concurrent if the statin prescription occurred prior to peak CPK; otherwise, they were coded into the daptomycin-alone group. Statistical analysis was conducted using SAS version 9.4 (SAS, Cary, NC).

## RESULTS

**Figure 1.**
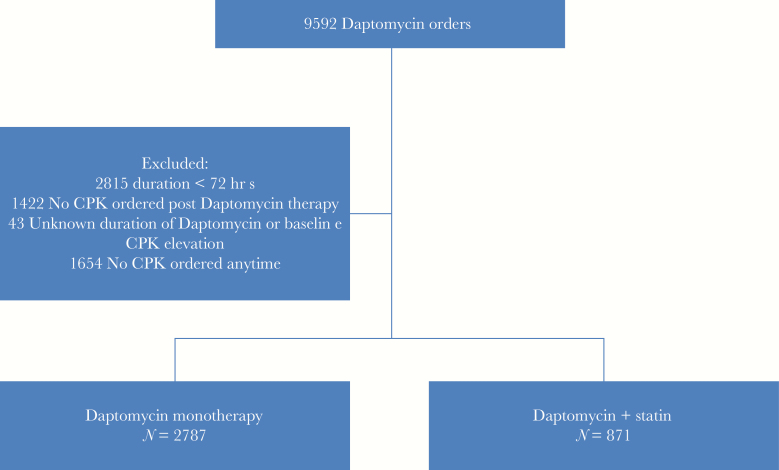
Flow Chart for Included Patients

Between April 2004 and April 2016, 9592 patients were identified as having been prescribed daptomycin therapy, of which 3658 were included in the study. Excluded patients can be found in the flow chart (Figure 1). Patients with baseline elevated CPKs were excluded as the elevation would not be attributable to the addition of daptomycin and is likely due to another confounding variable. Of the 3658 patients used for final analysis, 2964 had baseline CPK, whereas 694 did not have baseline CPK but had 2 CPKs while on therapy. The 694 patients who did not have a baseline CPK prior to daptomycin had an initial nonelevated CPK and a subsequent follow up CPK while on daptomycin. These patients were retained in the study as the initial CPK was not elevated. Of the 3658 patients, 2787 (76.2%) received daptomycin alone, and 871 (23.8%) received statins in addition. Summary statistics of demographic and other clinical characteristics of patients can be found in Table 1.

**Table 1.** Demographic and Clinical Characteristics of Eligible Patient

Patient Characteristics	Daptomycin group N = 2787	Concurrent statin group N = 871	*P* value
Age, mean, (standard deviation)	59.2 (16.3)	66.2 (12.7)	<.001
Male, n (%)	1346 (48.5%)	478 (54.9%)	.001
Race			.236
Caucasion	2147 (77%)	662 (76%)	
African American	496 (17.8%)	177 (20.3%)	
Other	144 (5.2%)	32 (3.7%)	
Elevated body mass index (%)	448 (16.1%)	183 (21.0%)	.001
Transplant (%)	100 (3.6%)	9 (1.0%)	<.001
Surgery (%)	1528 (54.8%)	474 (54.4%)	.834
Diabetes (%)	823 (29.5%)	431 (49.5%)	<.001
Cancer (%)	1043 (37.4%)	264 (30.3%)	<.001
Myocardial infarction (%)	103 (3.7%)	103 (11.8%)	<.001
Chronic kidney disease (%)	1181 (42.2%)	454 (52.1%)	<.001
Myalgia (%)	191 (6.9%)	56 (6.4%)	.663
Rhabdomyolysis (%)	0 (0%)	0 (0%)	1.00
Baseline creatine phosphokinase (%)	2255 (80.9%)	709 (81.4%)	.748
Elevated peak creatine phosphokinase (%)	90 (3.2%)	26 (3%)	.236

Of the total patients, the average age was 60.8 years and statistically different between groups. The concurrent statin group was older, with an average age of 66.2 years old compared with 59.2 years old in the daptomycin group. The concurrent statin group also had more male patients (54.9%) when compared with the daptomycin group (48.5%). The concurrent statin group had more patients with elevated BMI, diabetes, previous myocardial infarction, and chronic kidney disease. The daptomycin-alone group had more patients who had a history of cancer and transplantation.

Of the 871 patients receiving statins (in addition to daptomycin), 370 (42.5%) received atorvastatin, 238 (27.3%) received simvastatin, 180 (20.7%) received pravastatin, 49 (5.6%) received rosuvastatin, 17 (1.95%) received lovastatin, and 17 (1.95%) others received other statins. Statins dose ranged from 5 mg to 80 mg daily, with a modular dose being 20 mg to 40 mg. Eighty-one (9.3%) patients received 80 mg of the chosen statin.

**Figure 2.**
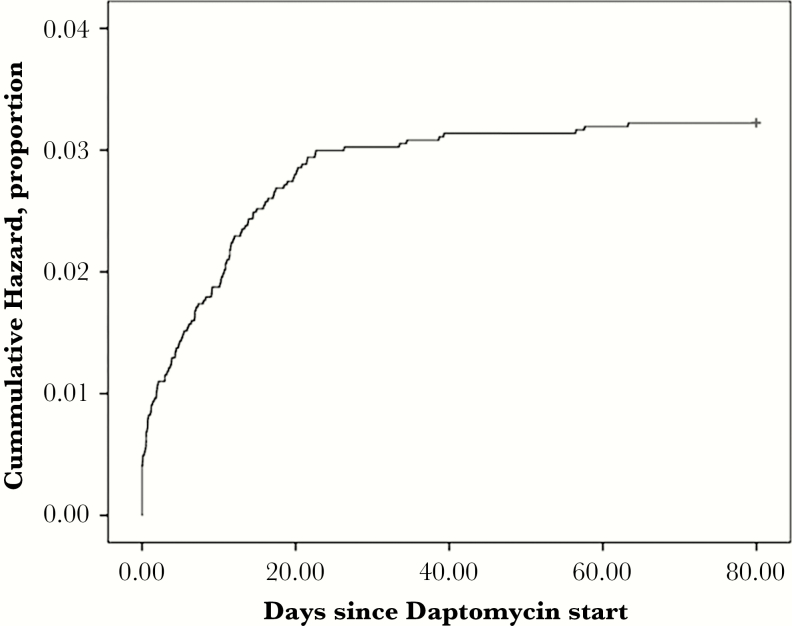
Kaplan-Meier Curve for Time to Peak Elevated Creatine Phosphokinase for All Patients

Out of the 3658 patients included in the study, 116 (3.2%) experienced a CPK elevation. The incidence of CPK elevation for the daptomycin group was 90 (3.2%), compared with 26 (3%) in the concurrent statin group. Out of these elevated CPKs, 88 occurred in the first week of daptomycin therapy and most of the events occurred by the 21^st^ day of therapy (Figure 2). The cumulative incidence of CPK elevation at days 7 and 21 were 2.8% and 3.0%, respectively. The last elevated CPK occurred on day 63, for a final cumulative incidence of 3.2%. The median number of CPKs monitored per patient was 3. Patients who received daptomycin therapy in addition to statins were 1.05 times as likely to experience CPK elevation compared with those on daptomycin alone (hazard ratio, 1.05; *P* = .85; 95% confidence interval, 0.61–1.84). After adjusting for potential risk factors, the hazards ratio remained similar (Table 2). Significant predictors of hazards of CPK elevation after controlling for other factors were African American race, BMI, and previous history of rhabdomyolysis prior to initiation of daptomycin (Table 2).

**Table 2.** Adjusted Hazard Ratio for Elevated Creatine Phosphokinase for All Patients (3658)

Variables	Hazard ratio	*P* value	95% confidence limit
Daptomycin and statin^a^	1.06	.857	0.60–1.88
Female	0.78	.188	0.53–1.15
Age	0.99	.581	0.98–1.01
Body mass index	1.03	.001	1.01–1.04
African American	2.18	.000	1.46–3.26
Other race	0.78	.791	0.11–5.63
Caucasian	1.00	—	—
Transplant	1.97	.15	0.79–4.88
Previous history of rhabdomyolysis	9.77	<.0001	4.71–20.29

^a^Time-varying covariate.

Statistical analysis was repeated after removing 694 (19%) patients who did not have baseline CPK values. After adjusting for potential risk factors, the hazard ratio remained the same for all patients, showing that there is no statistically significant association between statin and daptomycin therapy and CKP elevation.

Other factors explored, but which showed no statistical significance with respect to hazards of CPK elevation, were whether the patient had had surgery or a transplant, diabetes, or had a history of cancer, chronic kidney disease, or myalgia. There were no instances of rhabdomyolysis based on diagnosis codes that occurred during the study period in patients on daptomycin therapy in either group. A previous history of rhabdomyolysis was found in 8 patients in the concurrent statin group and 20 patients in the daptomycin group. A previous history of ever having documented rhabdomyolysis by ICD coding was associated with a hazard ratio of 9.77.

## DISCUSSION

To our knowledge, this is the largest study of the concomitant use of the daptomycin and statins and their effect on CPK. This study showed no statistical difference between the daptomycin group and the concurrent statin group. The trend was towards a slight increased event rate in the concurrent statin group. Although many of the patients were eliminated in the study due to the exclusion criteria, when the analysis was run with the subgroups included, the event rates were similar. After adjusting for sex, race, BMI, and comorbidity, there remained no statistical difference between groups.

Along with these findings, this study also showed a hazards ratio of 9.77 for all patients with a previous history of rhabdomyolysis. This is a concerning factor. It suggests that daptomycin should be avoided in patients who have ever had a diagnosis of rhabdomyolysis. As has been shown in previous studies, there was an increased risk of CPK elevation in the patients who were obese.

The literature associated with the concern of elevated CPK in patients on both daptomycin and statin therapy is small with mixed results. A matched case-control study and a few small retrospective case studies have been performed [4, 7, 8]. The second largest study after ours, a separate single-center matched case-control study with a total of 128 patients with CPK elevation, did show a statistically significant increase in CPK elevation in patients on daptomycin and statins. The cases were matched based on length of therapy. This study uses a clinically low CPK cutoff of only 200 U/L as a definition of myopathy, and this may lead to inclusion of patients with clinically insignificant CPK elevations [9]. A low CPK elevation may be normal in certain individuals depending on age, sex, and race [10]. We chose a CPK cut off of 1000 U/L as this aligns with the manufacturer’s recommendation to discontinue daptomycin therapy in patients with unexplained signs and symptoms of myopathy and a CPK >1000 U/L [3]. This study also had a very small number of patients analyzed that were on statins with only 46 patients total patients in both the cases and controls. There were only 32 patients in the group with CPK elevation ≥1 time the upper limit of normal and a total of 8 patients in the rhabdomyolysis (CPK elevation ≥10 times the upper limit of normal) group. Due to the small amount of patients evaluated on concurrent statins, the results may be underpowered. However, both the study described and the current study showed that obesity (BMI > 30) was associated with an increased risk of CPK elevation.

The remainder of the previous studies has failed to show a statistical difference in the incidence of CPK elevations for those receiving statin therapy with daptomycin versus daptomycin alone. Golightly et al studied the incidence of symptoms or objective muscle injury due to daptomycin with and without statins and found no significant statistical difference [4]. A previous study by Parra-Ruiz et al evaluated high dose daptomycin with and without statin use and showed an incidence of 8% CPK elevation (2.5 times the upper limit of normal) with combined daptomycin and statin therapy and 10% with daptomycin alone [11]. McConnell et al showed an incidence of 5.7% of CPK elevation with daptomycin and statin therapy versus 1.1% with daptomycin alone. This difference was not statistically significant as the number of total CPK elevation was only 5 patients [8]. Bland et al studied the effect of musculoskeletal outcomes on patients with both daptomycin and statins. Elevations of CPK were found in 10.2% of patients on combination therapy and in 5.3% of patients on daptomycin alone, which was not statistically significant. This study also looked at the incidence of myalgias between the 2 groups. The daptomycin-alone group had a myalgia incidence of 2.9%, and the combination group had an incidence of 6.1% [12]. The second largest published study completed was Berg et al. This retrospective study looked at a total of 498 patients. Although the direct incidence of each group was not listed, the patients receiving daptomycin and statin therapy concurrently were found to be almost twice as likely to experience an elevated CPK value when compare to daptomycin alone. This difference was not statistically significant [7]. Almost all of the studies showed an increased incidence of CPK elevations in the patients with concomitant daptomycin and statin therapy, but this increase was not statistically significant in a majority of studies. Most of the previous studies had a relatively small patient population. With multiple small studies not showing a statistical difference between groups and our larger study showing no statistical difference between groups, it is reasonable to conclude that the concomitant use of daptomycin and statins is reasonable.

The current recommendation that these agents are not to be used together comes from the concern for confounding CPK increase. Each agent has a separate mechanism leading to CPK increase. With statins, the risk of muscle injury has been related to genetic variations and drug interactions. This was studied specifically in simvastatin. In patient with SLCO1B1 polymorphism, the systemic exposure to simvastatin was significantly increased, which led to increased risk of muscle toxicity [13]. This same effect has been seen with pravastatin and pitavastatin as well [14]. In addition to genetic variants leading to muscle toxicity, there has been a link between drug-drug interactions with medications that are strong inhibitors of the CYP3A4, such as erythromycin [15]. The leading mechanism on statin-induced myopathy is thought to be secondary to abnormal mitochondrial function with depletion of CoQ10. It is thought that statins decrease mitochondrial function and protein synthesis based on multiple studies, which may lead to myopathy [16]. The mechanism behind daptomycin-related muscle injury is not completely understood. It is known that daptomycin is cleared by the kidneys and does not affect the CYP3A4 isoenzymes in the liver. A previous study by Bhavanani et al was able to demonstrate an associated increase in CPK within those with decreased creatinine clearance [1]. Animal models have suggested a link between daptomycin and sarcolemma damage [17, 18]. This animal model is thought to be the mechanism of muscle injury. The pathways of statin muscle injury and daptomycin muscle injury are not known to overlap.

As currently recommended, statins are held if a patient is in need of treatment with daptomycin. This does not come without risks. Statin withdrawal or rebound can lead to potentially fatal outcomes. Holding statin therapy has been shown to have negative cardiovascular outcomes in those with ischemic strokes and acute myocardial infarctions [5, 6, 19]. It is noted that rarely would a patient present in a situation with an acute coronary event and an infection needing daptomycin simultaneously. However, 1 study showed an association in holding statins and an increase in subarachnoid hemorrhages after discontinuation [20]. Also, in patients with a previous history of myocardial infarction, intermittent chronic statin therapy was associated with a higher mortality and decreased long-term survival [21, 22]. To avoid any possible risk of adverse effects, there is a need to determine if concomitant statin use with daptomycin causes an increase risk in CPK elevation and muscle injury.

The limitations of this study include the retrospective design that leads to potential incomplete inclusion bias. It was not possible to control for frequency and timing of CPK lab draws, which possibly led to missing values. As the data were obtained from pharmacy administration codes and electronic medical record databases, a manual chart review on each patient was not completed. This could possibly lead to missing CPKs from laboratories not associated with the electronic record. Also, we were not able to collect accurate daptomycin dosing for the patients. The advantages are that this study was done over different hospital settings, including both a large tertiary care center and multiple community hospitals, which may have different prescribing practices. Our study only included patients with follow up CPK values while on therapy, which differs from some of the published literature [4, 12].This is the largest series evaluating CPK elevations in patients on daptomycin and statins. There does not appear to be an increased risk of CPK elevation with the concomitant use of daptomycin and statins. Frequent CPK monitoring is needed in all patients on daptomycin. Further prospective studies are needed to clarify the findings of CPK elevations in the daptomycin and statin population. Clinicians may consider concomitant use of daptomycin and statin therapy with weekly CPK monitoring.
